# Hemodynamic effects of electrical muscle stimulation in the prophylaxis of deep vein thrombosis for intensive care unit patients: a randomized trial

**DOI:** 10.1186/s40560-016-0206-8

**Published:** 2017-01-13

**Authors:** Masahiro Ojima, Ryosuke Takegawa, Tomoya Hirose, Mitsuo Ohnishi, Tadahiko Shiozaki, Takeshi Shimazu

**Affiliations:** Department of Traumatology and Acute Critical Medicine, Osaka University Graduate School of Medicine, Suita, Osaka Japan

**Keywords:** Electrical muscle stimulation, Deep vein thrombosis, Hemodynamics, Thromboprophylaxis, Intensive care

## Abstract

**Background:**

Deep vein thrombosis (DVT) is a major complication in critical care. There are various methods of prophylaxis, but none of them fully prevent DVT, and each method has adverse effects. Electrical muscle stimulation (EMS) could be a new effective approach to prevent DVT in intensive care unit (ICU) patients. We hypothesized that EMS increases the venous flow of the lower limbs and has a prophylactic effect against the formation of DVT.

**Methods:**

This study included 26 patients admitted to a single ICU. We enrolled patients who could not move themselves due to spinal cord injury, head injury, central nervous system abnormalities, and sedation for mechanical ventilation. The patients were randomly allocated to either the EMS group or the control group. Patients in the EMS group received 30-min sessions of EMS applied to the bilateral lower extremities on arbitrary days within 14 days after admission. The control patients received no EMS. The peak flow velocity and diameter of the popliteal vein (Pop.V) and common femoral vein (CFV) were measured by ultrasound and then the volumes of venous flow were calculated using a formula.

**Results:**

There were no statistically significant differences in patient characteristics between the two groups except for the mortality rate. In the EMS group, the median and interquartile range (IQR, 25th–75th percentile) of velocities of the Pop.V and CFV were higher during EMS compared with at rest: 10.6 (8.0–14.8) vs 24.5 (15.1–37.8) cm/s and 17.0 (12.3–23.8) vs 24.3 (17.0–33.0) cm/s, respectively (*p* < 0.05). The median (IQR) of volumes of venous flow of the Pop.V and CFV at rest and during EMS were 4.2 (2.7–7.2) vs 8.6 (5.4–16.1) cm^3^/s and 12.9 (9.7–21.4) vs 20.8 (12.3–34.1) cm^3^/s, respectively (*p* < 0.05). There were no major complications related to EMS.

**Conclusions:**

EMS increased the venous flow of the lower limbs. EMS could be one potential method for venous thromboprophylaxis.

**Trial registration:**

UMIN000013642

## Background

Deep vein thrombosis (DVT) is one of the major complications in critical care and sometimes leads to fatal complication such as pulmonary embolism (PE). The in-hospital mortality rate associated with PE in 2010 in the USA was reported to be 4.4%, with 30-day and 6-month rates up to 9.1 and 19.6%, respectively [1]. To prevent accidental death by PE, it is important to prevent the formation of DVT of the lower limbs during the intensive care unit (ICU) stay.

Three factors are responsible for the formation of DVT: venous blood stasis, endothelial injury, and hypercoagulability [2]. Patients with trauma or severe diseases requiring mechanical ventilation are forced to be on long-term bed rest, which causes venous blood stasis of the lower limbs and puts them at high risk for DVT. It is well known that range of mobility exercises and early ambulation are important to prevent DVT. However, such exercises are difficult and sometimes impossible during intensive care. Most of the international guidelines recommend the use of intermittent pneumatic compression (IPC) or compression stockings, or early administration of anticoagulants to prevent DVT in high-risk patients [3]. However, these prophylactic methods cannot fully prevent DVT. A review suggested that critically ill patients commonly develop DVT with rates that vary from 10% to as high as 30% regardless of the prophylactic methods [4]. There are also several issues related to their use. IPC sometimes causes peroneal nerve palsy or compartment syndrome due to incorrect attachment [5], compression stockings sometimes cause hemodynamic complication through incorrect use [6], and anticoagulation involves a risk of major hemorrhage [7]. Thus, a more effective and safer approach to DVT prophylaxis is needed.

An electrical muscle stimulation (EMS) device has recently been used for the rehabilitation of immobilized people [8]. Circulating current between two electrodes generated by EMS causes cyclic contraction of muscles and results in rhythmic changes in venous blood flow, which is expected to have a prophylactic effect on DVT [9]. We have already shown that EMS prevented atrophy of muscles during prolonged bed rest [10]. Thus, we hypothesized that attaching EMS on the lower limbs might have a potential to produce a similar effect of exercise therapy even for the patients forced to be immobile for prolonged periods. Some reports suggest that EMS may have a preventive effect on DVT not only in healthy subjects [8] but also in total knee/hip arthroplasty patients [11, 12] and postoperative patients [13]. However, a report on EMS use in patients with major trauma failed to show a significant difference in the rate of DVT formation and venous flow parameters between the patients with and without EMS [14]. There are few reports on EMS aimed at the prophylaxis of DVT in critical care settings, so the effect of EMS on the lower limbs is controversial. Additionally, there are major differences between patients in ICU setting and patients in another clinical settings; critically ill patients often were not able to move themselves voluntarily and could not have communication with others. They also have not only general risk factors but also specific ICU risk factors of DVT, like sedation, strong analgesia, vasopressors, or central venous catheter. We think these differences may have some effects on EMS. We hypothesized that EMS might increase the venous flow of the lower limbs and might have a prophylactic effect for formulating DVT even in critically ill patients.

The purpose of this study was to evaluate the short-term effects of EMS on venous blood flow of the lower limbs in ICU patients by ultrasonography.

## Methods

### Enrollment

This study was approved by the institutional review board of Osaka University Hospital (No. 13361-3), an academic urban tertiary referral hospital in Suita, Japan. The study was conducted in the ICU of the emergency department of Osaka University Hospital from April 2014 to May 2015. The ICU has 17 beds and treated 885 patients in 2014. Formal written consent for participation in this study was obtained from each patient or their next of kin.

We enrolled patients who could not voluntarily move their lower limbs, that is, (a) patients with limitation of lower limb motion due to traumatic brain injury, spinal cord injury or cerebral infarction, or hemorrhage and (b) sedated patients who were on mechanical ventilation for more than 3 days. Patients were excluded if they were under 16 years of age, were pregnant and parturient women, suffered from cardiac arrest on admission, were implanted with material containing metal parts, were on a life-support device such as extracorporeal membrane oxygenation, had a history of neurological disorder or present evidence of venous thrombosis of the lower limbs, received treatment for subarachnoid hemorrhage, were complicated with congestive heart failure, were in an unstable general condition, had local infection at the sites of electrode application, or required rest of the lower limbs because of fracture. We included 26 patients in this study.

### Study protocol

The risk of forming DVT was evaluated by the guideline at Osaka University Hospital. Patients are stratified into the high-risk group if they had a previous history of DVT or PE, have an advanced cancer located in the pelvis, had hip/knee replacement surgery or spinal surgery, or have antiphospholipid antibody syndrome. The risk factors of forming DVT are as follows: age over 60, presence of cancer not including advanced cancer located in the pelvis, presence of inflammatory bowel diseases, suffering from congestive heart failure or acute myocardial infarction, suffering from cerebral infarction or spinal cord injury, presence of nephrotic syndrome, pregnant women, obesity with body mass index (BMI) over 30 kg/m^2^, oral contraceptive use, abnormality of clotting factors, tranexamic acid use, undergoing an operation in the dorsosacral position, undergoing thoracoscopic surgery, undergoing an operation of over 3 h in length, being forced to be on bed rest for over 3 days, or being immobilized in a plaster cast. Patients who had less than three risk factors were stratified into the low-risk group, those with three risk factors into the moderate-risk group, and those with four risk factors or more into the high-risk group.

Flowtron Excel (manufactured by ArjoHuntleigh, Malmö, Sweden) was used for IPC. Prevention of DVT by IPC was performed in the moderate- and high-risk patients if there were no contraindications such as arteriosclerosis obliterans. When a patient was evaluated as low-risk, the attending doctor decided to perform IPC or not. IPC was attached to the bilateral lower legs. The compression pressure was 40 mmHg. In this study, all patients were treated with IPC. Unfractionated heparin was also administered intravenously based on the attending doctor’s decision for the high-risk patients. The dose of heparin was adjusted by checking the activated partial thromboplastin time targeting 40 to 50 s. Compression stockings were not used in this study. The prevention of DVT was continued until the first ambulation or patient discharge. We assigned patients to the EMS group or the control group by drawing lots from a box containing 20 equally allocated lots.

### Electrical muscle stimulation

**Fig. 1:**
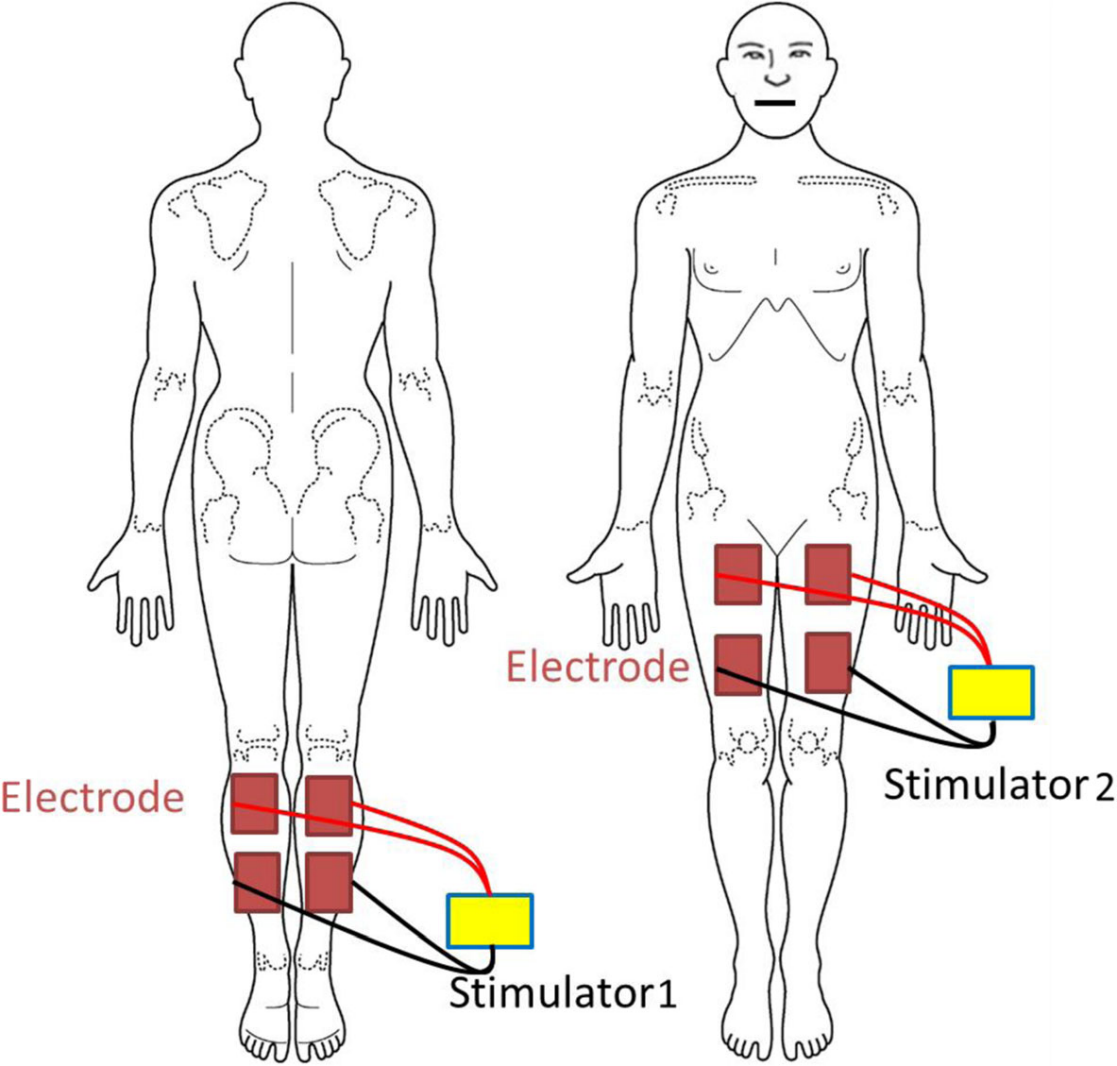
Position of the electrical muscle stimulation electrodes placed over the calf and quadriceps muscles

Patients in the EMS group were positioned in the supine position during EMS operation. We performed EMS only once a day on arbitrary days within 14 days after admission using the Torelete EM300 (distributed by Toray International, Inc., Tokyo, Japan; manufactured by ITO CO., LTD, Tokyo, Japan). EMS was performed at a time to suit researcher’s convenience. The average number of EMS implementation was 6.6 times per each case during study period (total EMS implementation was 93 times). Two pairs of EMS electrodes (PALS Platinum, Axelgaard Manufacturing Co., Ltd., Fallbrook, CA) were placed on the posterior calves and anteromedial thighs of both extremities to deliver electricity to the calf and quadriceps muscles (Fig. 1) with two EMS machines. Stimulation from the two machines was administered at the same time. The patients received one 30-min session on each experimental day. The duration of stimulation included 2 min for warm-up (pulse frequency 5 Hz, pulse width 150 μs), 26 min for training (50 Hz, 200 μs), and 2 min for cool-down (6 Hz, 150 μs). During the training time, muscle stimulation was set to a total cycle time of 19.2 s with an 8-s ON time, 8-s OFF time, 1.6-s ramp-up time, and 1.6-s ramp-down time. The output current of the device, which ranged from 20 to 50 mA, was set to obtain slight visual movement of the ankle in planter flexion. EMS was not conducted in the patients in the control group during the study period. IPC device was continuously activated between the EMS sessions in the EMS group.

### Doppler ultrasound measurements

**Fig. 2:**
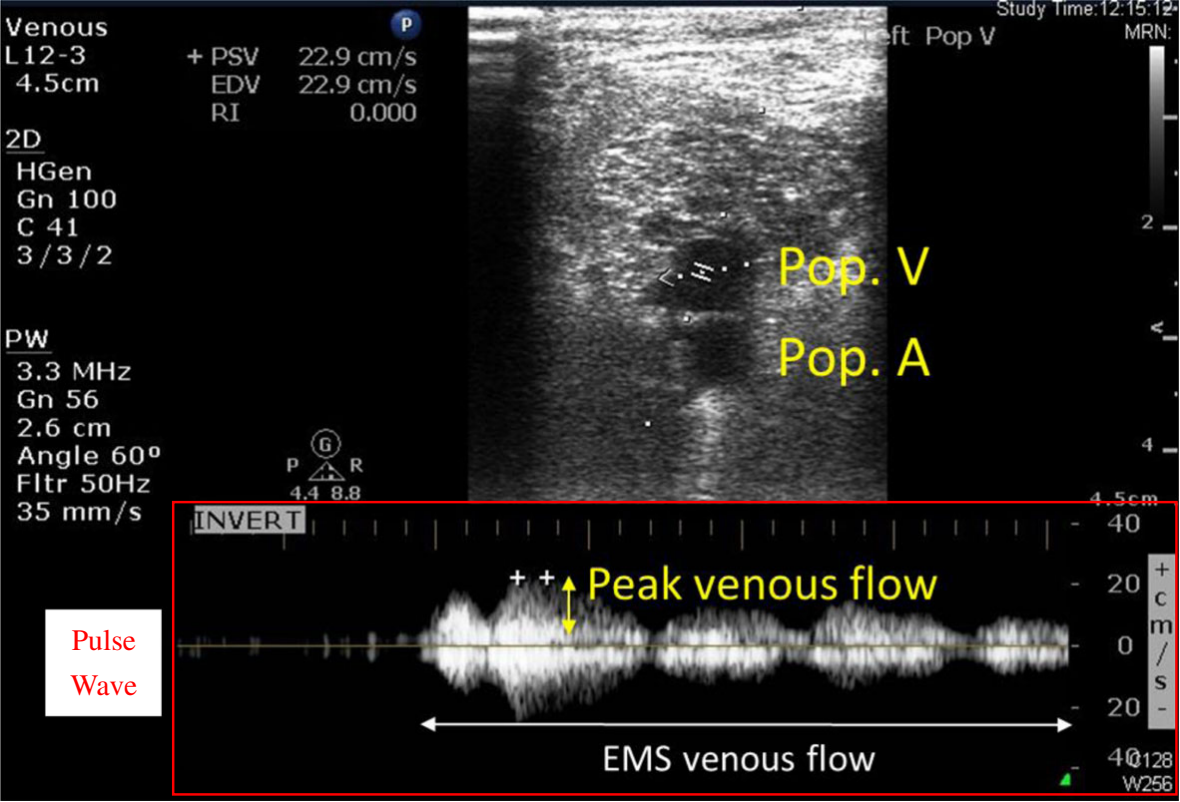
Representative screen shot from pulsed Doppler ultrasound for measurement of the peak venous velocity and diameter of the popliteal vein (Pop. V). Both were measured in the short-axis view. In this picture, the venous velocity was measured at the center of double line (denoted as *equal sign*) continuously and then the pulse wave was figured. The peak velocity was measured at the top of the pulse wave (denoted as *plus sign*)

The popliteal vein (Pop.V) and common femoral vein (CFV) were imaged in the short-axis view using a Philips CX50 CompactXtreme ultrasonic scanner (Philips Medical, Seattle, WA) and L12-3 broadband linear array transducer (Fig. 2). The measurements were taken at the popliteal fossa, just proximal to the venous confluence of the lower leg, and at the inguinal region, just below the inguinal ligament. The patients were positioned in the supine position with neutral positioning of the lower limbs when imaged of the CFV, while with externally rotated and mildly flexed knee when imaged of the Pop.V. In the EMS group, the peak venous blood flow velocity (cm/s) and the diameter (mm) of the Pop.V and CFV were recorded just before the application of EMS and 10 min after initiating EMS. The ultrasound probe was slightly angulated to face toward to obtain Doppler signal. The Doppler angle was fixed to 60 degrees during measurements of blood velocity. In the control group, the measurements were made similarly when the patient was at rest. All measurements were done by a single intensive care specialist well trained in ultrasound examination of the lower limbs.

After these measurements were obtained, we calculated the peak blood flow volume per second (cm^3^/s) in the Pop.V and CFV by the following equation:

Peak blood flow volume per second (cm^3^/s) = radius (cm) × radius (cm) × π (circular constant) × peak venous blood flow velocity (cm/s).

In this formula, “radius” refers to the radius of Pop.V or CFV, and “peak venous blood flow velocity” refers to the peak venous blood flow velocity of Pop.V or CFV.

### Patient data collection

Patient age, sex, height, body weight, BMI, Acute Physiology and Chronic Health Evaluation (APACHE II) score, Sequential Organ Failure Assessment (SOFA) score, Injury Severity Score (ISS), and diagnosis were recorded on admission. Each patient’s final outcome, the duration of mechanical ventilation, and side effects experienced with EMS were archived as well. We also confirmed the presence or absence of DVT by echography.

### Statistical analysis

Patient baseline demographic and clinical variables are presented as median and interquartile range (IQR, 25th–75th percentile). Data on patient sex, mortality, and presence or absence of sedation and analgesia were compared between the control group and EMS group using Fisher’s exact test. Data on the risk classification for DVT was compared between the control group and EMS group using Pearson’s chi-square test. Data on patient age, BMI, APACHE II score, SOFA score, and ISS were compared between the control group and EMS group using the Mann-Whitney *U* test. The differences in blood flow velocity, vessel diameter, and blood flow volume per second between the control group and the EMS group at rest and during EMS stimulation were analyzed using the Kruskal-Wallis test. Statistical analysis was performed using SPSS (version 22, SPSS, Chicago, IL). A *p* value of <0.05 was considered statistically significant.

## Results

Table 1 shows the patient characteristics of the two groups, which comprised 26 patients (52 legs, control group [*n* = 12], EMS group [*n* = 14]) and included 19 men and 7 women with a median (IQR) age of 70.0 (54.0–79.0) years. The diagnosis on admission was trauma in 12 patients, stroke in 6 patients, sepsis in 4 patients, and acute respiratory failure in 4 patients. Injuries in the 12 trauma patients were as follows: traumatic brain injury in 5, spinal cord injury in 5, pelvic injury in 1, and abdominal injury in 1. The median (IQR) of BMI, APACHE II score, SOFA score, and ISS on admission were 22.7 (20.9–24.5), 16.5 (9.0–22.0), 4.0 (2.0–5.0), and 25.0 (18.5–33.5), respectively. There were no statistical differences in these scores between the two groups. There was also no statistical difference in the probability of DVT between the two groups. The mortality rate was 19.2%, which was significantly higher in the control group.

**Table 1:** Patient characteristics in the control group and EMS group

	Control (*n* = 12)	EMS (*n* = 14)	*p* value
Age (years)	70.0 (61.0–79.0)	70.0 (51.0–79.0)	0.90
Sex (male/female)	9/3	10/4	1.00
Body mass index (kg/m^2^)	22.0 (20.2–24.2)	24.1 (21.3–28.1)	0.118
APACHE II score	20.0 (14.0–23.0)	12.1 (9.0–20.0)	0.27
SOFA score	4.0 (2.5–5.0)	2.5 (1.0–4.0)	0.40
ISS (*n* = 12)	25.0 (21.0–29.0) (*n* = 5)	25.0 (18.5–31.5) (*n* = 7)	0.76
Mortality (%)	5 (42)	0 (0)	0.012
Sedation (%)	11 (92)	10 (71)	0.33
Analgesia (%)	9 (75)	10 (71)	1.00
Admitting diagnosis			
Trauma	5	7	
Stroke	3	3	
Sepsis	2	2	
Others	2	2	
Risk classification for DVT			0.71
High risk	4	3	
Moderate risk	5	8	
Low risk	3	3	

Values are presented as median (IQR)

The peak blood flow velocity, venous diameter, and the peak blood flow volume per second of the Pop.V and CFV are shown in Table 2. The data in the EMS group was the summary of all the 93 sessions of EMS. The data in the control group was the result of the 66 times measurements. The peak venous flow velocities and peak blood flow volume of the CFV and Pop.V in both of the lower extremities were higher during EMS than at rest in the EMS group patients. The peak venous flow velocity and blood flow volume of Pop.V tended to be lower in the EMS group patients than in the control group patients in the resting state, but the differences were not statistically significant without the peak venous flow velocity of right Pop.V. The peak venous flow velocity of CFV tended to be lower in the EMS group patients than in the control group patients in the resting state, but the differences were not statistically significant. The blood flow volume of CFV was significantly lower in the EMS group patients than in the control group patients in the resting state. No differences were identified in venous diameter between the control group patients and the EMS group patients at rest or during EMS.

**Table 2:** Peak venous flow velocities, venous diameters, and peak blood flow volumes per second of Pop.V and CFV

	Control (*n* = 66)	EMS (*n* = 93)	
		At rest	During EMS
Peak venous flow velocity (cm/s)			
Right Pop.V	15.3 (11.3–26.0)	10.2 (7.6–14.0)^#^	24.3 (15.1–40.8)*^#^
Left Pop.V	13.3 (10.4–21.0)	10.9 (8.2–16.4)	24.9 (15.4–36.4)*^#^
Right CFV	20.4 (15.8–27.0)	16.3 (11.9–24.8)	25.1 (17.0–32.6)*
Left CFV	20.6 (16.2–28.6)	17.3 (13.8–23.3)	23.0 (18.2–34.3)*
Venous diameter (mm)			
Right Pop.V	6.8 (6.0–7.7)	6.9 (6.1–8.0)	7.3 (6.1–7.8)
Left Pop.V	6.9 (6.6–7.8)	7.0 (6.0–7.8)	7.2 (6.2–8.0)
Right CFV	11.0 (10.0–12.8)	10.2 (8.6–11.7)^#^	10.3 (9.2–11.7)
Left CFV	11.0 (9.8–12.7)	10.4 (9.2–12.0)	10.2 (8.9–12.0)
Blood flow volume (cm^3^/s)			
Right Pop.V	5.5 (3.6–9.0)	4.0 (2.5–7.2)	9.3 (5.3–14.7)*^#^
Left Pop.V	5.2 (3.4–8.2)	4.7 (2.9–7.2)	8.4 (5.5–16.9)*^#^
Right CFV	20.0 (12.4–35.0)	12.8 (9.4–19.6)^#^	20.8 (12.2–32.3)*
Left CFV	19.0 (12.7–35.4)	13.0 (10.7–22.4)^#^	20.9 (12.4–37.3)*

*Abbreviations: CFV* common femoral vein, *EMS* electrical muscle stimulation, *Pop.V* popliteal vein

Values are presented as median (IQR)

*Statistical difference compared with at rest in the EMS group patients (*p* < 0.05)

^#^Statistical difference compared with control patients (*p* < 0.05)

There were no major complications related to EMS. There were no changes of blood pressure, heart rate, and respiratory status during EMS (data not shown). No patients complained of discomfort from application of EMS. DVT and PE were detected in 1 patient in the control group, but no instances of DVT or PE occurred in the EMS group.

## Discussion

This is the first report, to our knowledge, to show the increase of venous flow in the lower extremities during EMS in the ICU setting by using ultrasound assessment. We showed that the peak venous velocity and volume of the CFV and Pop.V were significantly increased by EMS. These findings may indicate that EMS could be a new alternative for the prevention of DVT of the lower extremities.

We observed increases in peak venous velocity of nearly 2.2-fold in the Pop.V and 1.4-fold in the CFV with EMS compared to those in the resting condition. The maximum blood flow volume of the Pop.V rose by nearly 2.2-fold and that of the CFV rose by 1.5-fold with EMS compared to those in the resting condition. A previous report showed that IPC produces similar influences on velocity and volume of venous flow in the lower limbs [15]. It has been thought that EMS activates muscle pumping by contracting the lower extremity skeletal muscles and thus produces more physiological hemodynamic forces than IPC or compression stocking [16, 17]. From this point of view, one report compared EMS with IPC in terms of the influences of lower limb hemodynamics and showed that EMS led to more effective ejection of blood in conditions of venous stasis of the lower limbs [18]. It was shown that EMS had a potential for greater hemodynamic effect on the lower extremities than that of IPC. In addition, EMS did not influence the diameter of the Pop.V or CFV in this study. Another report addressed a similar finding in major trauma patients [14]. These findings imply that EMS intensifies the amount of venous return by activating muscle pumping without having a direct influence on major veins of the lower extremities.

There were a lot of EMS study, but most of them conducted EMS of calf muscles alone. Few reports addressing EMS effect with simultaneous stimulation of the thigh and calf muscles in clinical settings. A report was aimed to reveal EMS effect to reduce blood stasis during arthroplasty. The authors mentioned that EMS of calf muscles or thigh muscles alone was ineffective to put out venous-pooling blood from lower extremities to the central circulation [19]. We thought that simultaneous stimulation was important to maximize this effect and it would lead to prevent DVT more efficiently.

Blood flow velocity and the volume of Pop.V tended to be lower in the EMS group patients than in the control group patients in the resting state. These differences were possibly a result of the difference in BMI between the two groups: BMI values indicated that the EMS group patients were more obese than the control group patients. Obesity is one of the major risk factors for DVT [20]. Several reports have shown that venous return in the inferior vena cava or in the femoral vein is reduced in obese patients due to obesity-induced increases in intra-abdominal pressure [21, 22]. Our results were comparable with the results on venous flow of the lower extremities in these reports.

There were no major complications related to EMS, and it was well tolerated by the patients in this study. EMS is reported to be a relatively safe procedure to use in adults with advanced diseases, such as cancer or chronic obstructive pulmonary disease, and even in critically ill patients. Some reports also revealed that it did not affect the patient’s cardiorespiratory responses such as heart rate, blood pressure, oxygen saturation, and respiration rate. Compliance with its use is generally good, but it sometimes causes muscle discomfort, pain, or superficial burns, which may set limits to its use [23–25]. In intensive care settings, most of the patients are sedated and pain is controlled. This will relieve the pain associated with EMS and may make EMS more tolerable for ICU patients than non-ICU patients.

This study has several limitations. First, the efficacy of EMS to prevent DVT was not directly proven. High peak velocity and volume are not equal to better DVT protection. A large randomized controlled study is needed to elucidate this point. Second, blood inflow, i.e., arterial flow, in the lower extremities was not assessed in this study. It is possible for arterial flow to be influenced by EMS, which may have relevance to venous outflow. Arterial flow needs to be measured during EMS as well. Third, it was not clear which muscles should be stimulated, or when and how long and at what intensity muscles should be stimulated for DVT prophylaxis in ICU patients. These points also need to be assessed in future studies.

## Conclusions

EMS increased the venous flow of the lower limbs. This modality may have a prophylactic effect on DVT and could be one potential method for venous thromboprophylaxis, particularly in ICU patients. Further study is needed to confirm its optimal use.

## Abbreviations

APACHE: Acute Physiology and Chronic Health Evaluation
BMI: Body mass index
CFV: Central femoral vein
DVT: Deep vein thrombosis
EMS: Electrical muscle stimulation
ICU: Intensive care unit
IPC: Intermittent pneumatic compression
IQR: Interquartile range
ISS: Injury Severity Score
PE: Pulmonary embolism
Pop.V: Popliteal vein
SOFA: Sequential Organ Failure Assessment

## References

[1] Minges KE, Bikdeli B, Wang Y, Kim N, Curtis JP, Desai MM (2015). National trends in pulmonary embolism hospitalization rates and outcomes for adults aged ≥65 years in the United States (1999 to 2010). Am J Cardiol.

[2] Kumar DR, Hanlin E, Glurich I, Mazza JJ, Yale SH (2010). Virchow’s contribution to the understanding of thrombosis and cellular biology. Clin Med Res.

[3] Geerts WH, Pineo GF, Heit JA, Bergqvist D, Lassen MR, Colwell CW (2004). Prevention of venous thromboembolism: the Seventh ACCP Conference on Antithrombotic and Thrombolytic Therapy. Chest.

[4] Attia J, Ray JG, Cook DJ, Douketis J, Ginsberg JS, Geerts WH (2001). Deep vein thrombosis and its prevention in critically ill adults. Arch Intern Med.

[5] Lachmann EA, Rook JL, Tunkel R, Nagler W (1992). Complications associated with intermittent pneumatic compression. Arch Phys Med Rehabil.

[6] Collaboration CT, Dennis M, Sandercock PA, Reid J, Graham C, Murray G (2009). Effectiveness of thigh-length graduated compression stockings to reduce the risk of deep vein thrombosis after stroke (CLOTS trial 1): a multicentre, randomised controlled trial. Lancet.

[7] Eppsteiner RW, Shin JJ, Johnson J, van Dam RM (2010). Mechanical compression versus subcutaneous heparin therapy in postoperative and posttrauma patients: a systematic review and meta-analysis. World J Surg.

[8] Griffin M, Nicolaides AN, Bond D, Geroulakos G, Kalodiki E (2010). The efficacy of a new stimulation technology to increase venous flow and prevent venous stasis. Eur J Vasc Endovasc Surg.

[9] Stefanou C (2016). Electrical muscle stimulation in thomboprophylaxis: review and a derived hypothesis about thrombogenesis—the 4th factor. Springerplus.

[10] Hirose T, Shiozaki T, Shimizu K, Mouri T, Noguchi K, Ohnishi M (2013). The effect of electrical muscle stimulation on the prevention of disuse muscle atrophy in patients with consciousness disturbance in the intensive care unit. J Crit Care.

[11] Izumi M, Ikeuchi M, Aso K, Sugimura N, Kamimoto Y, Mitani T (2015). Less deep vein thrombosis due to transcutaneous fibular nerve stimulation in total knee arthroplasty: a randomized controlled trial. Knee Surg Sports Traumatol Arthrosc.

[12] Broderick BJ, Breathnach O, Condon F, Masterson E, Olaighin G (2013). Haemodynamic performance of neuromuscular electrical stimulation (NMES) during recovery from total hip arthroplasty. J Orthop Surg Res.

[13] Lobastov K, Barinov V, Laberko L, Obolensky V, Boyarintsev V, Rodoman G (2014). Electrical calf muscle stimulation with Veinoplus device in postoperative venous thromboembolism prevention. Int Angiol.

[14] Velmahos GC, Petrone P, Chan LS, Hanks SE, Brown CV, Demetriades D (2005). Electrostimulation for the prevention of deep venous thrombosis in patients with major trauma: a prospective randomized study. Surgery.

[15] Morris RJ, Woodcock JP (2004). Evidence-based compression: prevention of stasis and deep vein thrombosis. Ann Surg.

[16] Broderick BJ, O’Briain DE, Breen PP, Kearns SR, Olaighin G (2010). A pilot evaluation of a neuromuscular electrical stimulation (NMES) based methodology for the prevention of venous stasis during bed rest. Med Eng Phys.

[17] Lyons GM, Leane GE, Grace PA (2002). The effect of electrical stimulation of the calf muscle and compression stocking on venous blood flow velocity. Eur J Vasc Endovasc Surg.

[18] Broderick BJ, O’Connell S, Moloney S, O’Halloran K, Sheehan J, Quondamatteo F (2014). Comparative lower limb hemodynamics using neuromuscular electrical stimulation (NMES) versus intermittent pneumatic compression (IPC). Physiol Meas.

[19] Faghri PD, Van Meerdervort HF, Glaser RM, Figoni SF (1997). Electrical stimulation-induced contraction to reduce blood stasis during arthroplasty. IEEE Trans Rehabil Eng.

[20] Abdollahi M, Cushman M, Rosendaal FR (2003). Obesity: risk of venous thrombosis and the interaction with coagulation factor levels and oral contraceptive use. Thromb Haemost.

[21] Sterling SA, Jones AE, Coleman TG, Summers RL (2015). Theoretical analysis of the relative impact of obesity on hemodynamic stability during acute hemorrhagic shock. Arch Trauma Res.

[22] Willenberg T, Clemens R, Haegeli LM, Amann-Vesti B, Baumgartner I, Husmann M (2011). The influence of abdominal pressure on lower extremity venous pressure and hemodynamics: a human in-vivo model simulating the effect of abdominal obesity. Eur J Vasc Endovasc Surg.

[23] Williams N, Flynn M (2014). A review of the efficacy of neuromuscular electrical stimulation in critically ill patients. Physiother Theory Pract.

[24] Maddocks M, Gao W, Higginson IJ, Wilcock A (2013). Neuromuscular electrical stimulation for muscle weakness in adults with advanced disease. Cochrane Database Syst Rev.

[25] Segers J, Hermans G, Bruyninckx F, Meyfroidt G, Langer D, Gosselink R (2014). Feasibility of neuromuscular electrical stimulation in critically ill patients. J Crit Care.

